# UEMS training requirements for the speciality of psychiatry: knowledge, skills and professionalism required for the care of refugees and asylum seekers

**DOI:** 10.1192/j.eurpsy.2023.95

**Published:** 2023-07-19

**Authors:** M. Casanova Dias

**Affiliations:** ^1^Cardiff University, Cardiff, United Kingdom; ^2^Section of Psychiatry, UEMS (European Union of Medical Specialists), Brussels, Belgium

## Abstract

**Abstract:**

The UEMS (or European Union of Medical Specialists) is a non-governmental organisation representing 40 national associations of medical specialists and operating through 43 Specialist Sections. The Psychiatry Section’s purpose is to promote the highest standard of care for people who are affected by mental health problems in Europe through postgraduate training and continuing medical education of psychiatrists. It is responsible for developing the European Training Requirements (ETR) for the Specialty of Psychiatry through an iterative process that involved consulting national psychiatric associations, trainees, patient and carer organisations, EPA and WPA. Because medical education and the practice of psychiatry are continually evolving, it is intended that the ETR will be seen as a living document that will be periodically reviewed and amended.

In 2016, the Section approved an Annex to the competency framework covering learning outcomes of knowledge, skills and professionalism required for the care of refugees and asylum seekers. Last year, the Section undertook a major update and revision of the ETR and these learning outcomes have now been fully incorporated.

In this presentation, I am going to outline the specific requirements related to the care of refugees and asylum seekers. They relate to knowledge of traumatic stress, transcultural psychiatry and health promotion and social inclusion; intercultural communication skills, detailed history taking with reference to the impact of adverse events and psychoeducational skills; and professionalism, including awareness of own world view, respecting culture, ethnic and religious difference.

**Disclosure of Interest:**

None Declared

